# "Test and treat" or presumptive treatment for malaria in high transmission situations? A reflection on the latest WHO guidelines

**DOI:** 10.1186/1475-2875-10-136

**Published:** 2011-05-20

**Authors:** Bertrand Graz, Merlin Willcox, Thomas Szeless, André Rougemont

**Affiliations:** 1 Institute of Social and Preventive Medicine, University of Geneva, Switzerland; 2 Department of Primary Health Care, University of Oxford, UK

## Abstract

Recent WHO guidelines recommend a universal "test and treat" strategy for malaria, mainly by use of rapid diagnostic test (RDT) in all areas. The evidence for this approach is questioned here as there is a risk of over-reliance on parasitological diagnosis in high transmission situations, which still exist. In such areas, when a patient has fever or other malaria symptoms, the presence of *Plasmodium *spp neither reliably confirms malaria as the cause of the fever, nor excludes the possibility of other diseases. This is because the patient may be an asymptomatic carrier of malaria parasites and suffer from another disease.

To allow clinicians to perform their work adequately, local epidemiologic data are necessary. One size does not fit all. If parasite prevalence in the population is low, a diagnostic test is relevant; if the prevalence is high, the test does not provide information of any clinical usefulness, as happens with any test in medicine when the prevalence of the tested characteristic is high in the healthy population. It should also be remembered that, if in some cases anti-malarials are prescribed to parasite-negative patients, this will not increase selection pressure for drug resistance, because the parasite is not there.

In high transmission situations at least, other diagnoses should be sought in all patients, irrespective of the presence of malaria parasites. For this, clinical skills (but not necessarily physicians) are irreplaceable, in order to differentiate malaria from other causes of acute fever, such as benign viral infection or potentially dangerous conditions, which can all be present with the parasite co-existing only as a "commensal" or silent undesirable guest.

## Background

The latest WHO guidelines on the treatment of malaria state that, whenever possible, "*in all settings, clinical suspicion of malaria should be confirmed with a parasitological diagnosis*" [1,2]. This is a significant change from the previous guidelines [3], which recognized that parasitological diagnosis is not always necessary, particularly in high transmission areas. This new universal "test and treat" recommendation is based on a series of questionable assumptions (Table 1) [1]. The question of whether to test and treat or to treat presumptively is still hotly debated [4,5].

**Table 1:** Advantages and disadvantages of parasitological diagnosis

Advantages of parasitological diagnosis (according to WHO guidelines, section 6.1, 6.2)	Disadvantages of parasitological diagnosis in high transmission areas
Diagnosis based on clinical features alone has very low specificity and results in over-treatment	Parasitological diagnosis has low specificity

Improved patient care in parasite-positive patients	In a patient with fever, the presence of parasites neither reliably confirms malaria as the cause of the fever, nor excludes the possibility of other diseases

Identification of parasite-negative patients in whom another diagnosis must be sought	Other diagnoses should be sought **in all patients**, irrespective of the presence of malaria parasites

Prevention of unnecessary use of anti-malarials, reducing frequency of adverse effects especially in those who do not need the medicines, and drug pressure selecting for resistant parasites	Clinicians often prescribe anti-malarials even for patients with a negative test. Prescribing anti-malarials to parasite-negative patients will not increase selection pressure for new drug resistant mutations.

Improved malaria case detection and reporting	Some "cases" detected in high-transmission areas are incidental carriers of malaria parasites, presenting with another disease

Confirmation of treatment failures	RDTs cannot confirm treatment failures. It is only possible to do this with microscopy.

WHO's assumptions are discussed one by one, considering their applicability to high-transmission settings, which still exist. Basically, the « level of transmission » in malarial areas is an entomological concept. In most cases, the so-called transmission level is derived from the proportion of infected people in the general population. For practical purposes, it is considered here that « low transmission » is characteristic of areas where this proportion in the general population is below 20%. Between 20 and 50%, one can speak of « moderate transmission » and « high » above 50%. Since there is no universal definition, or cut-off figures, for "high-transmission", the discussion below will detail at what levels of parasite prevalence in the population a parasitological test supports, or does not support, clinical decisions.

## Review of the evidence for the assumptions underlying WHO's recommendation

### Assumption 1: "*diagnosis based on clinical features alone has very low specificity and results in over-treatment*"

The specificity of a diagnostic algorithm or test almost always varies according to the prevalence of the target condition. Clinical diagnosis and parasitological tests for malaria are no exception. The specificity of clinical diagnosis of malaria varies according to age group, time, place and the epidemiology of the disease. In low-transmission settings, specificity of clinical diagnosis is low and specificity of parasitological tests (e.g. rapid diagnostic tests - RDTs) is high, whereas in high-transmission areas the reverse is the case[6]. In children in a high-transmission setting, during the rainy season, high fever of short duration, with no other obvious cause, is most likely to be malaria [7]. In some such settings over 80% of febrile patients (all ages) with presumed malaria are parasite positive, and the "test and treat" strategy is not cost-effective, particularly in children and where the cost of treatment is lower than that of the test [8,9].

### Assumption 2: "*patient care will be improved in parasite-positive patients*"

This assumption is incorrect, particularly in high transmission areas. In a randomized controlled trial (in Burkina Faso in 2006), comparing outcomes in patients treated either presumptively or after use of an RDT, there was no difference in outcomes between groups[10], i.e. the use of RDT did not improve care in this setting. The more prevalent a characteristic is in the healthy population, the less useful it is to test for this characteristic as a means of detecting disease.

**Figure 1:**
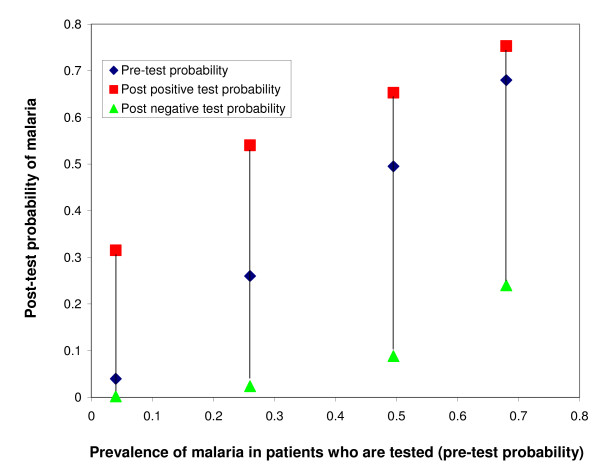
**Pre-test and post-test probability of infection with malaria parasites for patients with a positive or negative test result, in areas of differing malaria prevalence, assuming a sensitivity and a specificity of the test of 95%**. (Data from[11])

**Figure 2:**
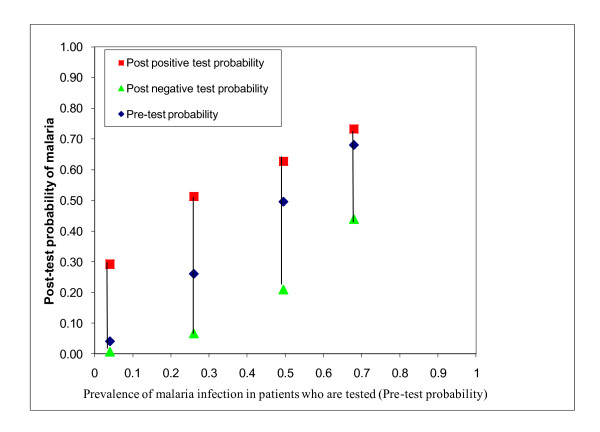
**Pre-test and post-test probability of infection with malaria parasites for patients with a positive or negative test result, in areas of differing malaria prevalence, assuming a sensitivity of 85% and a specificity of the test of 95%**. (Data from[11])

A useful tool for evaluating the clinical utility of a test is to calculate the likelihood ratio of having a disease, based on a positive or a negative result. The likelihood ratio multiplied by the pre-test odds of the disease gives the post-test odds. Figures 1, and 2 show how pre- and post-test probability of malaria infection for a negative and a positive test result change according to the background prevalence of infection with malaria parasites and the sensitivity of the test, 95% in Figure 1 and 85% in Figure 2 (based on statistical modelling[11]). The 85% sensitivity might be closer to what happens when the test has been stored in relatively hot conditions [12].

**Figure 3:**
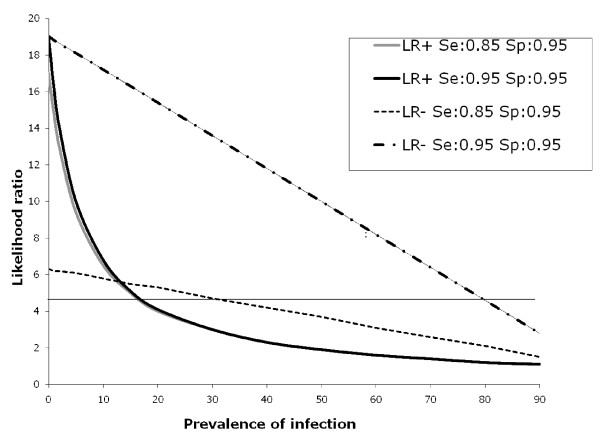
**Likelihood ratios of malaria when the parasite detection test is positive (LR+) and likelihood ratios of the absence of malaria when the parasite detection test is negative (LR -), according to the prevalence of infection in the general population, and to the sensitivity and specificity of the test for the detection of parasites**. Clinically useful tests are above the line (LR > 5). (Data from [11])

In summary, where the prevalence of malaria infection is low, a parasitological test is useful: if it is negative, the post-test probability of malaria is almost zero. If the test is positive, the probability of malaria is greatly increased. However at higher levels of prevalence, a negative test does not rule out infection with malaria parasites, and a positive test does not greatly increase the probability of malaria infection (which is already high). There is no clear threshold for applying a cut-off, but one commonly used rule is that a test is clinically useful to "rule in" or "rule out" a disease if the positive likelihood ratio is > = 5, or if the negative likelihood ratio is < = 0.2 respectively[13]. Applying this rule, a positive test ceases to be clinically useful at a prevalence of > 20% (see figure 3).

Parasite prevalence has declined in some parts of Africa, but the median in a recent review is still 22%[14]. Of note this review did not include any studies from rural West Africa after 2000, and excluded community-based studies. Table 2 shows the prevalence of malaria in populations where diagnostic tests have been deployed since 2005. The weighted overall mean parasite prevalence ( = pre-test probability) was 51.7% (of 87 703 patients tested). Populations develop partial immunity against malaria by the age of five years and in such semi-immune individuals even high levels of parasite density do not reliably predict fever incidence [15].

**Table 2:** Prescribing behaviour of clinicians for parasite-negative patients in different contexts, 2005-2010

% of parasite-negative patients receiving any antimalarial	Total N of parasite-neg pts	% of parasite-negative patients receiving antibiotics	Diagnostic test	Country	Setting	Transmission level	% of patients positive for malaria	Year of study	Ref
4.0%	5162	35.6%	RDT	Tanzania	Rural Health Centres	high	51.5%	2008	[28]

9.0%	14777		Microscopy and RDT	Uganda	District hospital outpatients	high	45.0%	2010	[29]

11.7%	700	45.0%	RDT	Zanzibar	Primary Health Care	high	30.0%	2005	[21]

27.0%	247	50.0%	Microscopy	Tanzania	Hospital Outpatients	high	38.4%	2005	[22]

30.0%	218	50.0%	RDT	Tanzania	Hospital Outpatients	high	38.4%	2005	[22]

31.8%	4661		RDT	Uganda	Primary Health Care	Low - high	625%	2007	[39]

35.5%	183		RDT	Zambia	Outpatients, rural and urban	high	44.2%	2006	[30]

36.0%	392		RDT	Kenya	Government health facilities	Low - high	12%	2006	[40]

40.4%	141	55.3%	RDT	Tanzania	Hospital Outpatients	high	55.4%	2006	[41]

46.0%	1298	28.8%	RDT	Ghana	Rural health centres	high	26.2%	2007-2008	[20]

49.5%	1013	35.0%	RDT	Ghana	Rural health centres	high	37.7%	2007-2008	[20]

49.5%	1325	28.6%	Microscopy	Ghana	Rural health centres	high	26.9%	2007-2008	[20]

54.6%	416	50.0%	Microscopy	Tanzania	Hospital Outpatients	low	0.4%	2005	[22]

58.0%	401	50.0%	RDT	Tanzania	Hospital Outpatients	low	0.4%	2005	[22]

58.4%	77		Microscopy	Zambia	Outpatients, rural and urban	high	45.4%	2006	[30]

61.3%	68	87.1%	RDT/microscopy	Kenya	Outpatients > = 5 years	high	52.7%	2006	[42]

63.0%	367	50.0%	Microscopy	Tanzania	Hospital Outpatients	low-moderate	8.3%	2005	[22]

63.0%	386	50.0%	RDT	Tanzania	Hospital Outpatients	low-moderate	8.3%	2005	[22]

75.0%	52	84.6%	RDT/microscopy	Kenya	Outpatients < 5 yrs	high	50.0%	2006	[42]

79.8%	287	54.7%	RDT	Burkina Faso	Primary care, rural, dry season	low	28.2%	2006	[10]

82.6%	206	59.9%	RDT	Burkina Faso	Primary care, rural, rainy season	high	68.2%	2006	[10]

In a patient with fever (or other malaria symptoms) in a high-transmission area, the presence of parasites neither reliably confirms malaria as the cause, nor excludes the possibility of other diseases [9]. Like many commensal organisms, *P. falciparum *can produce symptoms, but may also be asymptomatic (it may, however, have undesirable effects in the long run). Detecting the presence of parasites can be misleading. It may divert the attention of clinicians from other diagnoses if they do not conduct an appropriate clinical examination. For example, the case of an infant is reported with a positive malaria test who was only treated with anti-malarials but subsequently died of presumed pneumonia[10]. Paradoxically the care of parasite-positive patients may worsen through over-reliance on diagnostic tests at the expense of clinical examination [4].

### Assumption 3: "*identification of parasite-negative patients in whom another diagnosis must be sought*"

In high transmission areas, other diagnoses should be sought **in all patients**, irrespective of the presence of malaria parasites. Some analyses assume that there is no co-infection between malarial and bacterial infections[16], but this assumption is clearly incorrect. For example 14% of unconscious children aged < 1 year presenting to Kilifi hospital in Kenya had both malaria parasites and definite bacterial meningitis[17]. Therefore in secondary care settings (in high-transmission areas), in children presenting with serious febrile illness, bacterial illness should be considered regardless of parasitological test results [18].

However it is inappropriate to extrapolate findings from severely ill children in secondary care settings, to children with uncomplicated febrile illness in primary care settings[5], which are the majority of the cases in which parasitological tests have been used (Table 2). A parasite-negative child with uncomplicated febrile illness is *not *at high risk of other life-threatening bacterial diseases, and the most common other diagnosis is viral upper-respiratory tract infection [9,19]. Unless other clinical features of a bacterial infection are present, antibiotics are not indicated.

Antibiotics should not be overused in primary care settings, otherwise there is a higher risk of evolution and spread of resistant bacteria (because all human beings harbour commensal bacteria which can evolve resistance to antibiotics, and become pathogenic). Some studies show that use of RDTs increases antibiotic prescribing in malaria-negative patients [20,21] (Table 2). If the disease is a common cold, antibiotics should generally be avoided. For example, in hospital outpatients in Tanzania, 74% of children aged < 5 years with a negative malaria test were prescribed an antibiotic[22]. This is not necessarily an indicator of good practice. In another trial, this time in Uganda, 45% of children with upper respiratory tract infections and common colds were prescribed an antibiotic [19]. The need for antibiotics or other treatments should be determined by clinical history and examination, not by the presence or absence of malaria parasites. Key for this is the local quality of clinical work and teaching. Indeed there is an interesting parallel between the decision to prescribe anti-malarials and the decision to prescribe antibiotics for chest infections. The latter is often based on presumptive diagnosis from clinical features [23], and a "bacterial test" would be of dubious utility because there are many healthy carriers.

The utility of a negative result also depends on the sensitivity of the test [11] (see Figures 1-2). In practice, sensitivity of RDTs varies widely according to setting [12] and brand[24]. Although some studies have shown excellent sensitivity[25], others have shown that sensitivity is much lower in field conditions[12]; this can be due to storage temperatures above 30 degree Celsius, for example. Sensitivity of RDTs is lower for low levels of parasitaemia[9,26]. These are irrelevant in semi-immune patients (age > 5 years in high-transmission settings) and so in these patients it is safe not to treat RDT-negative patients with anti-malarials[27]. However in non-immune infants even very low parasitaemia cause disease and many of these are missed by RDTs, which is particularly important in high transmission situations[26]. Of course, the sensitivity of clinical diagnosis varies as well, but in a high-transmission setting presumptive treatment of all febrile infants with anti-malarials is probably safer than relying on the result of an RDT[26].

### Assumption 4: "*prevention of unnecessary use of anti-malarials, reducing frequency of adverse effects, especially in those who do not need the medicines, and drug pressure selecting for resistant parasites*"

Many studies found that diagnostic testing did not prevent the use of anti-malarials for patients in whom no parasites are detected. Table 2 summarizes studies of prescriptions for parasite-negative patients since 2005. The two largest and most recent studies in Tanzania and Uganda found that only 4% and 9% of parasite-negative patients were prescribed anti-malarials, although in Tanzania the test-and-treat strategy was only used for patients aged 5 years and older [28,29]. Other studies found that much larger proportions of malaria-negative patients still received anti-malarials. The weighted mean of all the studies (for a total of 87703 patients) was that 23.3% of parasite-negative patients received a prescription for anti-malarials. In summary, the use of RDTs reduced prescribing of anti-malarials in some studies [21,28-30] but in several cases, using RDTs significantly increased costs to the health service provider[31]. In no case was there any evidence of clinical benefit to patients or of improved cost-effectiveness. The argument that treating malaria-negative patients will increase the drug pressure for resistant parasites seems logically incoherent because malaria-negative patients harbour no parasites. Long-acting drugs given together with artemisinin derivatives have a long, slow tail phase of drug elimination, during which time new infections may be acquired. Parasites which already have resistance to these drugs may be selected for, but it is improbable that any new resistant mutations would develop in this context because the biomass of parasites in the inoculum is low. A strategy for preventing resistance could be to keep ACTs for patients at highest risk of severe malaria (non-immune patients) and to use other medicines for semi-immune patients (aged 5 years and older, in high-transmission areas), who will improve with other treatments[32].

### Assumption 5: "*improved malaria case detection and reporting*"

Parasitological diagnosis will enable health information systems to report more accurately whether patients consulting with fever or treated for malaria actually had evidence of being parasitaemic. For example, in Senegal, there has been a large decrease in reported malaria cases, in part due to better case ascertainment with RDTs [30]. However, it is not clear whether this advantage alone justifies the extra expense of systematically making a parasitological diagnosis. Regular population studies might be more appropriate for health information and policy design.

While case detection may be improved in low-transmission areas, in high-transmission areas what will often be detected and reported is incidental parasitaemia (the presence of malaria parasites in a patient presenting symptoms not attributable to the malaria parasite) rather than clinical malaria. A more useful measure is the population attributable fraction of fever due to malaria[26]. Furthermore, since the results may vary considerably in the same individual on the same day, the usual single blood test to determine the presence or absence of parasites may be misleading [33,34]. In short, parasitological diagnosis does not mean case detection, because of the importance of carriers who are not cases. However, RDTs may well be the tool of choice for rapid assessment of local epidemiology.

### Assumption 6: "*Confirmation of treatment failures"*

Rapid diagnostic tests based on detection of the HRP-2 antigen often remain positive for over five weeks after the disappearance of live parasites, because they detect the HRP-2 antigen which is still present in debris from dead parasites for some time after total parasite clearance [35]. This also results in a high "false positive rate" and low specificity of RDTs compared to microscopy, particularly in high transmission areas [6,36]. If RDTs are used to "confirm" treatment failures, many patients will be given a second unnecessary treatment.

**Figure 4:**
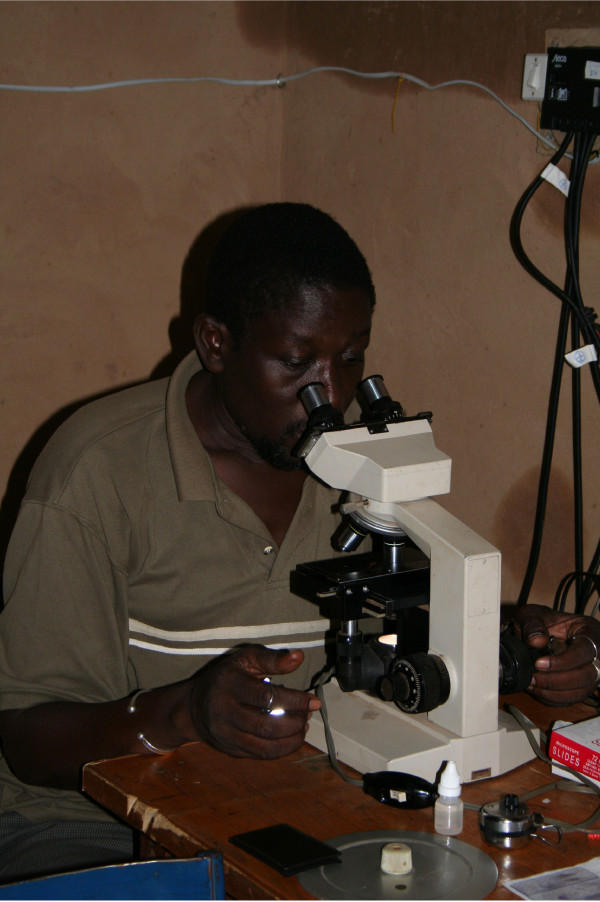
**Fagnan Sanogo performs microscopy for malaria parasites in a research clinic in Mali**. High-quality microscopy is rarely sustained outside the research setting (Photo by M. Willcox).

Microscopy does not help to confirm treatment failure unless it is of excellent quality, which is rare outside of research settings. In such settings, when high-quality microscopy is available, a negative film is very good at detecting the absence of malaria parasites in the blood (Figure 4) and it is unusual for a patient with a negative blood film (made and read according to a high quality protocol, with double or triple readings of every slide) to subsequently develop malaria [19]. However, it is rarely possible to sustain such high quality microscopy outside the research setting, and poor quality microscopy is not clinically useful [37,38] for confirmation of treatment failures.

## Discussion

From the review of the evidence above it seems that a universal "test and treat" strategy is neither evidence-based nor cost-effective. The new WHO guidelines can be understood in the context of the long term goal of elimination of malaria. However, this is not achievable in the foreseeable future in many areas. WHO's assumptions justifying parasitological diagnosis may well be correct in low-transmission settings, but not in high-transmission settings. The prevalence of malaria parasitaemia has declined in some parts of Africa, so these areas are transitioning from high-transmission to low-transmission. In these areas, diagnostic tests will become increasingly important. However, many areas of sub-Saharan Africa remain high-transmission areas, and the appropriateness and cost-effectiveness of universal testing in these areas is questionable.

Malaria is a complex disease and it is impossible to write clinically useful global guidelines on its management, which do not take local epidemiology into account. In practice countries contain areas with differing levels of transmission, which also depends on seasonality. It would make more sense to adapt the strategy according to local factors. This is the way health professionals deal with any infectious disease (evaluating the probability of a particular disease case by case, according to season, age, place of residence, recent travel, and so on). It is perfectly possible to apply two different policies simultaneously (for example presumptive treatment for patients aged < 5 years and test-and-treat for older patients)[28]. Even where such a policy does not formally exist, health workers are more likely to prescribe anti-malarials for RDT-negative patients aged < 5 years than for those aged 5 years and over[39]. If health care workers are able to do better than blindly applying global guidelines, this should not be considered "second class" treatment. The level of skill required to identify serious infection, malaria and 'trivial' viral infection is probably achieved through standard medical training in most countries, but it remains to be seen to what extent it is also achievable for shorter training in other health professions.

If the WHO recommendations have little influence on clinical practice, this may not be due solely to problems of policy implementation, but also to the fact that the debate has been closed too early[1]. Those clinicians using their clinical skills and not following the recommended "test and treat strategy" in high transmission areas do not increase the danger for their patients nor jeopardize the future of the malaria global strategy. They may well act this way not against their will or because of logistical problems or lack of information. The reliance on clinical skills and "bedside reasoning", based on, among others, the knowledge of local epidemiology, makes good sense in the clinical encounter. The presumptive treatment strategy for malaria could be in the best interest of some patients, now and in the future, at least in high-transmission areas where eradication is not in sight.

Cost-effectiveness must also be an important consideration especially in settings where health care resources are limited. In settings of high transmission the cost saving from avoiding ACT is outweighed by the greater cost of deploying RDTs universally [9,31]. RDTs are not cost-effective even in low-transmission areas, unless adherence to their results is high [8]. Even where the adherence is relatively good there is no clear evidence of any clinical benefit[20]. There may be higher priorities for limited resources than universal parasitological diagnosis.

## Conclusion

There is a need for policies and clinical strategies adapted to different settings. Each area should develop its own guidelines based on sound evidence, taking into consideration both the local epidemiology of malaria and other febrile diseases and a realistic assessment of available healthcare resources. It would be a dangerous mistake to rely too much on diagnostic tests in areas where parasite prevalence is high. Clinical examination and reasoning remain necessary in order to differentiate malaria from other causes of acute disease such as benign viral infection or potentially dangerous conditions (which can all be present with the parasite co-existing only as a commensal or undesirable guest). To allow clinicians to perform their work adequately, local epidemiological data are necessary: in areas of low parasite prevalence, a diagnostic test is relevant; if the prevalence is high, the test does not provide information of any clinical usefulness. In this latter case, clinical skills are irreplaceable.

## Consent

Written informed consent was obtained from all individuals for publication of accompanying images.

## Conflict of interest

The authors declare that they have no competing interests.

## Authors' contributions

BG and AR wrote a first draft of the article which was substantially revised by MW and TS. All authors read and approved the final manuscript.
